# Immune Transcriptional Response in Head Kidney Primary Cell Cultures Isolated from the Three Most Important Species in Chilean Salmonids Aquaculture

**DOI:** 10.3390/biology12070924

**Published:** 2023-06-28

**Authors:** Daniela P. Nualart, Francisco Dann, Ricardo Oyarzún-Salazar, Francisco J. Morera, Luis Vargas-Chacoff

**Affiliations:** 1 Fish Physiology Laboratory, Institute of Marine and Limnological Sciences, Faculty of Sciences, Universidad Austral de Chile, Valdivia 5090000, Chile; franciscojavierdann@gmail.com; 2 Ph.D. Program in Aquaculture Sciences, Universidad Austral de Chile, Puerto Montt 5480000, Chile; 3 Millennium Institute Biodiversity of Antarctic and Subantarctic Ecosystems, BASE, University Austral of Chile, Valdivia 5090000, Chile; 4 Centro Fondap de Investigación de Altas Latitudes (IDEAL), Universidad Austral de Chile, Valdivia 5090000, Chile; 5 Laboratorio Institucional, Facultad de Ciencias de la Naturaleza, Universidad San Sebastián, Puerto Montt 5480000, Chile; ricardo.oyarzun@uss.cl; 6 Applied Biochemistry Laboratory, Institute of Pharmacology and Morphophysiology, Faculty of Veterinary Sciences, Universidad Austral de Chile, Valdivia 5090000, Chile; fjmorera@uach.cl; 7 Integrative Biology Group, Universidad Austral de Chile, Valdivia 5090000, Chile

**Keywords:** immune response, head kidney cells, primary culture, LPS, POLY I:C, salmonids

## Abstract

**Simple Summary:**

Protecting the welfare of animals and the 3Rs rule are very relevance, and for this reason cell culture is an important tool. In fish it has importance in several fields such as virology, toxicology, pathology and immunology. The objective was to carry out a primary culture of the head kidneys of Atlantic salmon (*Salmo salar*), Pacific salmon (*Oncorhynchus kisutch*) and rainbow trout (*Oncorhynchus mykiss)*, and to characterize how they respond to bacterial and viral stimuli by analyzing molecules that participate in the innate and adaptive immune response. The primary cell cultures of the head kidney (HK) from the three salmonids studied were cultured and exposed to two substances that mimic molecular patterns of different pathogens, i.e., Lipopolysaccharide (LPS) and Polyinosinic: polycytidylic acid (POLY I:C). The HK primary cell cultures from the three species grown in vitro responded differently to POLY I:C and LPS. This is the first study to demonstrate and characterize the expression of immune genes in head kidney primary cell cultures isolated from three salmonid species. It also indicates their potential role in developing immune responses as defense response agents and targets of immunoregulatory factors.

**Abstract:**

Fish cell culture is a common in vitro tool for studies in different fields such as virology, toxicology, pathology and immunology of fish. Fish cell cultures are a promising help to study how to diagnose and control relevant viral and intracellular bacterial infections in aquaculture. They can also be used for developing vaccines and immunostimulants, especially with the ethical demand aiming to reduce and replace the number of fish used in research. This study aimed to isolate head kidney primary cell cultures from three Chilean salmonids: *Salmo salar*, *Oncorhynchus kisutch*, and *Oncorhynchus mykiss*, and characterize the response to bacterial and viral stimuli by evaluating various markers of the innate and adaptive immune response. Specifically, the primary cell cultures of the head kidney from the three salmonids studied were cultured and exposed to two substances that mimic molecular patterns of different pathogens, i.e., Lipopolysaccharide (LPS) (bacterial) and Polyinosinic: polycytidylic acid (POLY I:C). Subsequently, we determined the mRNA expression profiles of the TLR-1, TLR-8, IgM, TLR-5, and MHC II genes. Head kidney primary cell cultures from the three species grown in vitro responded differently to POLY I:C and LPS. This is the first study to demonstrate and characterize the expression of immune genes in head kidney primary cell culture isolated from three salmonid species. It also indicates their potential role in developing immune responses as defense response agents and targets of immunoregulatory factors.

## 1. Introduction

Chile is among the ten countries with the highest aquaculture production worldwide (FAO 2022 [1]). Almost 70% of this activity corresponds to the cultivation of three Salmonid species, Atlantic salmon (*Salmo salar)*, Pacific salmon (*Oncorhynchus kisutch),* and rainbow trout (*Oncorhynchus mykiss*) [2]. Unfortunately, these species in Chile [3] are affected by stressful conditions and various pathogens, which can reduce growth and affect their health, having a high economic impact on aquacultural activity and environmental health, as happened during March 2021 in the Chilean fjords [4].

Bacteria, viruses, and most immunostimulants are recognized as pathogen-associated molecular patterns (PAMPs) [5] and are detected by pattern recognition receptors (PRRs) on the immune cells [6,7]. The main families of PRRs include Toll-like receptors (TLRs), Nod-like receptors (NLRs), RIG-like receptors (RLRs), AIM2-like receptors (ALRs), and C-type lectin receptors [8,9].

Toll-like receptors (TLRs) are outspread in extracellular, membrane and cytoplasmic compartments, are classified according to their ligand specificity, function and cellular localization and play an essential role in innate immunity by recognizing different conserved microbial motifs, carbohydrates, peptides, lipopolysaccharides, and lipoproteins and glucans or endogenous substances collectively known as microbial-/pathogen-/danger-associated molecular patterns (MAMPs/PAMPs/DAMPs) [10].

In teleosts, more than 20 TLRs have been identified with an important role in providing the first line of defense in fish [11]. These receptors are localized on the cell surface such as TLR-1, TLR-2, TLR-4, and TLR-5, while others, such as TLR3, TLR-7, TLR-8 and TLR9, are found in the membrane of intracellular compartments, such as endosome, lysosome or endolysosome [11,12]. These TLRs are expressed in the membrane of B cells, macrophages, and dendritic cells [13] and can interact with Polyinosinic: polycytidylic acid (POLY I:C), which is used to simulate viral infections. POLY I:C is structurally similar to double-stranded RNA and can be recognized by TLR-3 and TLR-8 [14]. In addition, Lipopolysaccharide (LPS) is the main component of the outer membrane of gram-negative bacteria and is recognized by TLR1, TLR4 and TLR5 [14,15]. Therefore, LPS and POLY I:C are commonly used for scientific research on the fish immune system as bacterial and viral stimuli, respectively [16].

On the other hand, there are histocompatibility molecule receptors that are expressed only by professional antigen-presenting cells, dendritic cells (DCs), B cells, and macrophages, where antigens are loaded and transferred to the cell surface on the major histocompatibility complex (MHC class II) [17,18]. The antigen presentation with MHC class II to T-cell receptors (TCRs) activates naïve T cells and induces the differentiation of T cells into different helper T-cell subsets based on the antigen, thus mediating the T-cell response [11,17,18,19,20,21]. Their binding with the respective PAMPs triggers intracellular signaling pathways that lead to the production and release of proinflammatory cytokines and components of the adaptive system, including T-cell receptors (TCR) and immunoglobulins (Igs) [19,20].

The cellular components of the innate and adaptive immune systems of fish include a varied diversity of cells, for example an MHC II ((MHC class II) which plays a vital role in exogenous and endogenous antigens, and also which is only expressed in the professional antigen-presenting cells (pAPCs), which mainly populate lymphoid organs including the head kidney and spleen to subsequently engulf pathogens, and display peptides processed from exogenous antigens via lysosomal pathways [18]. On the other hand, the adaptive immune components include immunoglobulins, which are expressed on the B cells’ surface as B-cell receptors or in a soluble form in body fluids, mucosal cells, gut [22] and gills [23] and are the main constituents of the immune response against pathogens. IgM can be expressed at the surface of B cells [11,24], and is the most predominant immunoglobulin in fish serum, which can activate mechanisms involving humoral immunity mediated by IGs such as pathogen elimination via phagocytosis and toxin and virus neutralization, and complement cascade activation [25].

Animal welfare is becoming increasingly more relevant, and the use of animals is being limited and must be regulated using, for example, the “3Rs rule” (Replace, Reduce, Refine). Cell culture is highly relevant as it minimizes animal usage and has been instrumental in studying viral and microbial pathogens in humans and animals [26,27]. An increasing number of specific in vitro methods have been developed for fish [28,29,30], including primary fish gill and liver culture [31] and intestinal epithelial cells [32]. These can be initiated from explants or enzymatic dissociation, require less adaptation to culture media, are economical and represent more closely what physiologically occurs in the host [29,30], allowing the study of more specific responses between species as in this work.

This is the first study of a primary culture of head kidney cells from explants, where the molecular markers of the innate and adaptive immune system are compared and established in the three commercially important salmonid species facing a challenge with immunostimulants. This study aimed to isolate head kidney primary cell cultures from the three main salmonids produced by Chilean aquaculture: Atlantic salmon (*Salmo salar*), Pacific salmon (*Oncorhynchus kisutch*), and rainbow trout (*Oncorhynchus mykiss*), and characterize their immune response to bacterial and viral stimuli by evaluating various markers that encode core molecules of the innate and adaptive immune response. All these primary culture cells were exposed to LPS (bacterial stimuli) and POLY I:C (viral stimuli) to subsequently determine the mRNA expression profiles of TLR-1, TLR-5, TLR-8, IgM, and MHC II as gene markers of an immune response.

## 2. Materials and Methods

### 2.1. Animals

Healthy specimens of rainbow trout (*O. mykiss*), Atlantic salmon (*S. salar*), and Pacific salmon (*O. kisutch*), approximately weighing 200.32 ± 12.4 g, were obtained from a fish farm and were transported to laboratories at the Faculty of Science (Universidad Austral de Chile, Valdivia). Three fish of each salmon species were sampled (*O. mykiss*, *S. salar*, and *O. kisutch*), and each tissue had three replicates. All fish were captured, anesthetized with a lethal dose of 2-phenoxyethanol (1 mL/L, Fluka-77699-500ML), and euthanized by spinal sectioning before tissue removal [33].

All experimental protocols complied with guidelines for the use of laboratory animals, as established by the Chilean National Commission for Scientific and Technological Research (ANID) and the Universidad Austral de Chile.

### 2.2. Head Kidney Primary Cell Culture (HKPCC) Preparation

For the primary culture, we obtained small pieces of tissue (approximately 10 mg) or explant of the head kidney from *O. mykiss*, *O. kisutch,* and *S. salar* under aseptic or sterile conditions [34], which were then seeded and kept in a six-well plate and grown at 18 °C under air atmosphere for at least 72 h. The cell- and tissue-growth medium was Leibovitz’s 15 (L-15) supplemented with 10% fetal bovine serum (FBS) (Invitrogen) and 1% penicillin-streptomycin (P/S) (Gibco, Thermo Fisher, Waltham, MA, USA).

### 2.3. In Vitro Immunostimulation

24 h after seeding the explant for a head kidney primary cell culture, it was stimulated with LPS (Invivo Gen#BSS-40-01) or POLY I:C (Invivo Gen#PIC-40-04) using the same concentrations (LPS: 10 ng/mL); POLY I:C: 10 ng/mL) previously described to study the effect of these immunostimulants on innate immune defense [13]. Primary cell cultures in six well plates were exposed to the immunostimulants for 0.5, 1, 3, 6, 12, 24, and 48 h at 18 °C. Control plates had the same volume of medium without the immunostimulant. All experiments were run in triplicate and independently repeated twice.

### 2.4. Total RNA Extraction

Total RNA was isolated from stimulated and control HKPCC using TRIzol reagent (Sigma) following the manufacturer’s instructions and stored at −80 °C. Subsequently, RNA was quantified at 260 nm on a NanoDrop spectrophotometer (NanoDrop Technologies^®^), and the quality was determined by electrophoresis on a 1% agarose gel. Finally, total RNA (2 μg) was used as a reverse transcription template to synthesize cDNA, applying MMLV-RT reverse transcriptase (Promega) and the oligo-dT primer (Invitrogen) according to standard procedures.

### 2.5. qRT-PCR Analysis of Gene Expression

Reactions were carried out on an AriaMx Real-time PCR System (Agilent). cDNA was diluted to 100 ng and used as a qRT-PCR template with reactive Brilliant SYBRGreen qPCR (Stratagene). Reactions were performed in triplicate, in a total volume of 14 μL, which contained 6 μL SYBRGreen, 2 μL cDNA (100 ng), 1.08 μL of primer mix, and 4.92 μL of PCR-grade water. The applied PCR program was as follows: 95 °C for 10 min, followed by 40 cycles at 90 °C for 10 s, 60 °C for 15 s, and 72 °C for 15 s. Melting curve analysis of the amplified products was performed after each PCR to confirm that only one PCR product was amplified and detected. Expression levels were analyzed using the comparative Ct method (2^−ΔΔCT^) [35]. The data are presented as the fold change in gene expression normalized to an endogenous reference gene (18S) and relative to unstimulated cells (control). The primers used for TLR-1, TLR-5, TLR-8, major histocompatibility complex class II (MHCII), and immunoglobulin M (IgM) were obtained from [36] and are listed in Table 1. PCR efficiencies were determined by linear regression analysis of sample data using LinRegPCR [37] from the serial dilutions when Log dilution was plotted against DCT (threshold cycle number).

### 2.6. Statistical Analysis

Significant differences in gene expression between different treatments were determined by two-way analysis of variance (two-way ANOVA).

All data are shown as the mean ± standard error (SE). Assumptions of normality and homogeneity were tested for the detected variances. Differences were evaluated using one-way ANOVA and were considered significant at *p* < 0.05 and followed by Tukey’s test (*p* < 0.05).

## 3. Results

### 3.1. Morphology of Head Kidney Culture Cells

**Figure 1:**
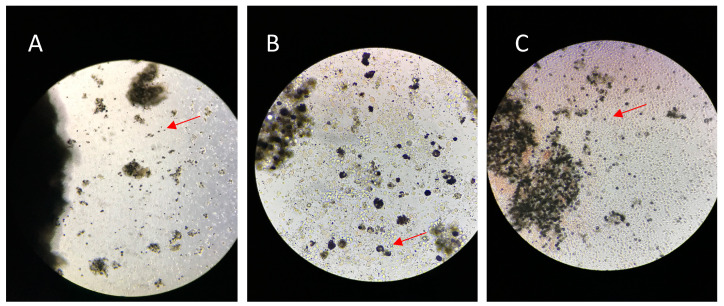
Morphology of head kidney primary culture cells (10×). (**A**) HKPCC from *S. salar*; (**B**) HKPCC from *O. kisutch*; (**C**) HKPCC from *O. mykiss*. The arrows indicate the cells of the primary culture.

The head kidney primary cells seeded were observed migrating along with the tissue after 24 h; where they exhibited significant heterogeneous cell populations, irregular shapes, some of the adherent type and, to a lesser extent, cells in suspension at 18 °C incubation (Figure 1), then treatment with LPS and POLY I:C was started.

### 3.2. mRNA Gene Expression Changes

#### 3.2.1. TLR-1 Expression

**Figure 2:**
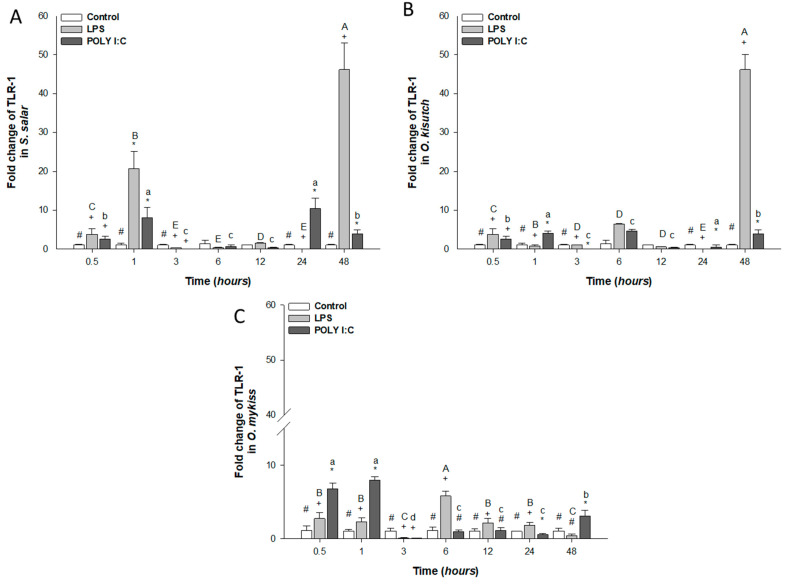
Gene expression of TLR-1 in the head kidney primary cell culture (HKPCC) stimulated by LPS and POLY I:C. (**A**) *S. salar*, (**B**) *O. kisutch* and (**C**) *O. mykiss*. Each value represents the mean ± S.E.M. (n = 3). Uppercase letters indicate statistical differences in LPS over time, and lowercase letters are for POLY I:C statistical differences over time-points. Symbols (*, # and +) indicate statistical differences among different treatments (control, LPS and POLY I:C) at the same time-point (two-way ANOVA, *p* < 0.05).

mRNA gene expression of TRL-1 in head kidney cells stimulated with LPS increased significantly at 1, 3 and 48 h in *S. salar* and *O. kisutch* (Figure 2A,B), and statistical differences between treatments with POLY I:C and control cells were observed.

In *O. mykiss*, TLR-1 transcription in LPS-stimulated cells was increased throughout the experimental period, but at 3, 24 and 48 h, there was a significant decrease compared to the control cells. TLR-1 mRNA significantly increased in *S. salar* and *O. kisutch* at 1, 24, and 48 h in HKPCC exposed to POLY I:C, but this decreased significantly between treatments at times 3, 6 and 12 h. In *O. mykiss*, increased gene expression was observed at 0.5, 1, and 48 h. (Figure 2C).

#### 3.2.2. TLR-5 Expression

**Figure 3:**
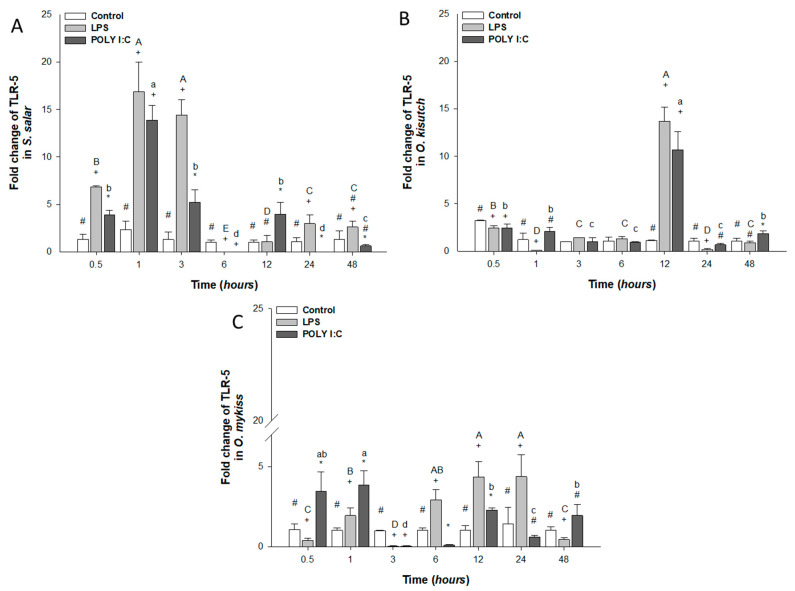
Gene expression of TLR-5 in the head kidney primary cell culture (HKPCC) stimulated by LPS and POLY I:C. (**A**) *S. salar*, (**B**) *O. kisutch* and (**C**) *O. mykiss*. Each value represents the mean ± S.E.M. (n = 3). Uppercase letters indicate statistical differences in LPS over time, and lowercase letters are for POLY I:C statistical differences over time-points. Symbols (*, # and +) indicate statistical differences among different treatments (control, LPS and POLY I:C) at the same time-point (two-way ANOVA, *p* < 0.05).

TLR-5 mRNA expression was significantly up-regulated at 0.5, 1, 3, and 24 h in *S. salar* when exposed with LPS (Figure 3A) and between treatments with POLY I:C. However, in *O. kisutch*, mRNA increased at 1, 6 and 12 h with both stimuli (Figure 3B). In *O. mykiss*, high TLR-5 expression was observed at 1, 6, 12, and 24 h when exposed to LPS and 1, 12 and 48 h for POLY I:C (Figure 3C). Moreover, when stimulated with POLY I:C, the cell response decreased significantly at 6, 24, and 48 h in *S. salar* and *O. mykiss*. mRNA expression was maintained from 3 h to 12 h in *O. kisutch* when these cells were treated with LPS and POLY I:C. Throughout the stimulation kinetics, significant differences were observed between cells treated with LPS, POLY I:C and control cells.

#### 3.2.3. TLR-8 Expression

**Figure 4:**
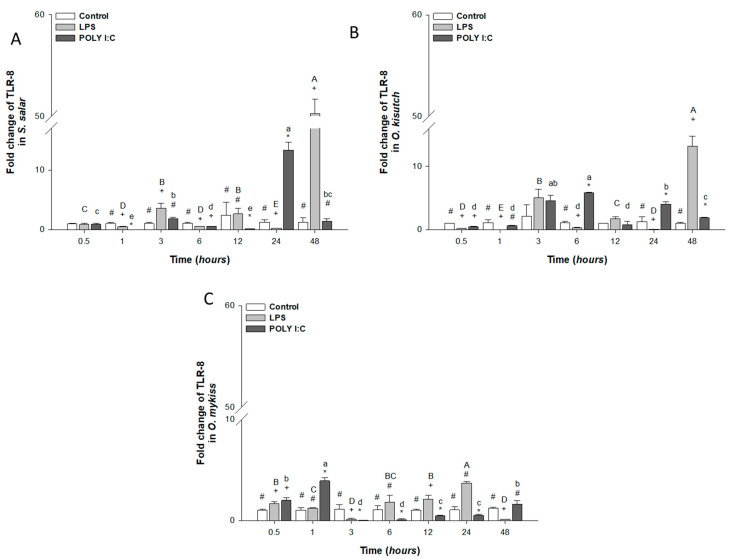
Gene expression of TLR-8 in the head kidney primary cell culture (HKPCC) stimulated by LPS and POLY I:C. (**A**) *S. salar*, (**B**) *O. kisutch* and (**C**) *O. mykiss*. Each value represents the mean ± S.E.M. (n = 3). Uppercase letters indicate statistical differences in LPS over time, and lowercase letters are for POLY I:C statistical differences over time-points. Symbols (*, # and +) indicate statistical differences among different treatments (control, LPS and POLY I:C) at the same time-point (two-way ANOVA, *p* < 0.05).

TLR-8 mRNA expression increased significantly after exposure to LPS at 3, 12 and 48 h in *S. salar and O. kisutch* (Figure 4A,B, respectively). Meanwhile, the expression increased significantly in *O. mykiss* at 0.5, at 12 and 24 h (Figure 4C) with respect to the control. However, in *O. mykiss,* decreased gene expression was observed at 3 and 48 h compared to the control. In the case of *S. salar* and *O. kisutch,* the drops were at 1, 6, and 24 h.

In the case of POLY I:C stimulation, HKPCC from *S. salar* presented a significant increase in TLR-8 mRNA only at 24 h. Meanwhile, HKPCC from *O. kisutch* and *O. mykiss* presented increases at 3, 6, and 24 (6.8-fold increase) and 0.5 and 1 h, respectively (Figure 4A,B). However, in *O. mykiss,* this gene was downregulated significantly from 3 to 24 h (Figure 4C) and in *S. salar* at 1, 6, and 12 h, compared to the unstimulated control cells and POLY Y I:C. It was also possible to observe throughout the kinetics of stimulation; significant differences were observed between cells treated with LPS, POLY I:C and control cells.

#### 3.2.4. IgM Expression

**Figure 5:**
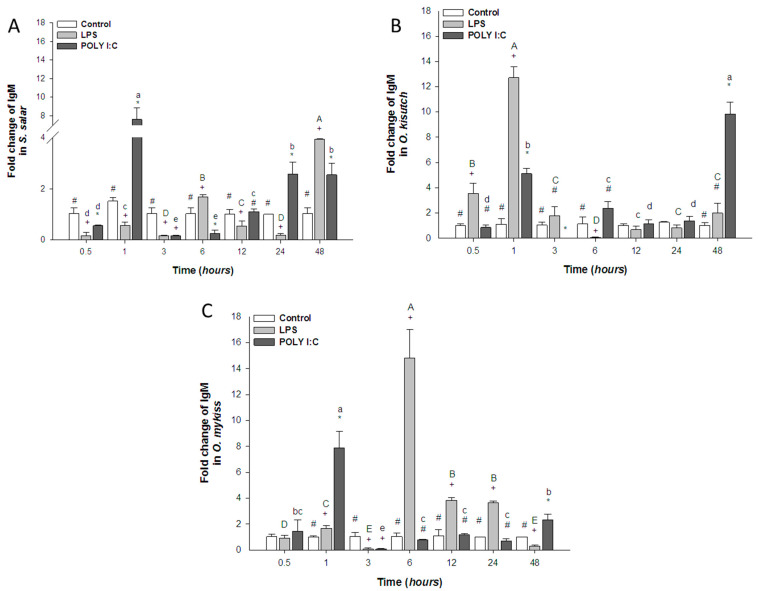
Gene expression of IgM in the head kidney primary cell culture (HKPCC) stimulated by LPS and POLY I:C. (**A**) *S. salar*, (**B**) *O. kisutch* and (**C**) *O. mykiss*. Each value represents the mean ± S.E.M. (n = 3). Uppercase letters indicate statistical differences in LPS over time, and lowercase letters are for POLY I:C statistical differences over time-points. Symbols (*, # and +) indicate statistical differences among different treatments (control, LPS and POLY I:C) at the same time-point (two-way ANOVA, *p* < 0.05).

After LPS exposure, IgM mRNA expression increased significantly at 6 and 48 h in *S. salar* (Figure 5A), at 0.5 to 1 h in *O. kisutch* (Figure 5B), and at 6, 12, and 24 h in *O. mykiss* (Figure 5C) compared with the control cells and POLY I:C.

When stimulated with POLY I:C, head kidney cells from *S. salar* (Figure 5A) increased IgM expression at 1, 24, and 48 h and at 1, 6, and 48 h in *O. kisutch* (Figure 5B). In *O. mykiss* cells, IgM expression was decreased at 3 and 24 h and increased at 1 and 48 h (Figure 5C). Differences were observed between treatments throughout the kinetics with the exception of *S. salar* at 0.5 h.

#### 3.2.5. MHC II Expression

**Figure 6:**
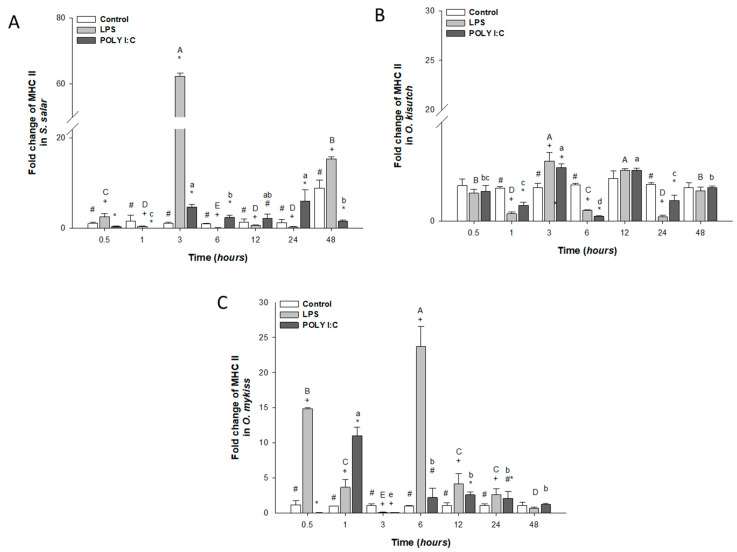
Gene expression of MHCII in the head kidney primary cell culture (HKPCC) stimulated by LPS and POLY I:C. (**A**) *S. salar*, (**B**) *O. kisutch* and (**C**) *O. mykiss*. Each value represents the mean ± S.E.M. (n = 3). Uppercase letters indicate statistical differences in LPS over time, and lowercase letters are for POLY I:C statistical differences over time-points. Symbols (*, # and +) indicate statistical differences among different treatments (control, LPS and POLY I:C) at the same time-point (two-way ANOVA, *p* < 0.05).

MHC II mRNA expression increased significantly after exposure to LPS in *S. salar* at 3 and 48 h (Figure 6A) and in *O. mykiss* at 0.5, 1, 6, and 12 h (Figure 6C). However, in *O. kisutch* (Figure 6B), a sustained response was observed during all kinetics as compared to the HKPC control cells treated with both immunostimulants.

In *S. salar* HKPCCs stimulated with POLY I:C, expression was up-regulated at 0.5 and 1 h (Figure 6A), but in *O. mykiss* cells, the increase was observed at 1 and 12 h (Figure 6C). In contrast, down-regulation of MHC II was observed at 0.5 and 3 h (Figure 6C). Throughout the stimulation kinetics, significant differences were observed between cells treated with LPS, POLY I:C and control cells.

## 4. Discussion

Fish do not have bone marrow, and the head kidney is the primary lymphoid tissue in fish that produces blood cells. Therefore, it plays a vital role in innate and acquired immune responses in fish [8,24]. It produces the first response according to the signal, being TLRs the primary barrier (for example TRL1, TRL5, and TRL8) and the antibodies such as IgM (IgM+ B cells comprise the majority of B cells in tissues such as the head kidney) are the secondary barrier. Innate immunity has an essential role in protecting fish from endogenous and exogenous pathogenic invasion. Meanwhile, adaptive immunity is a more specific barrier [18,32]. For this reason, to have primary cell culture as the standard method to reduce the number of fish employed in research is very important, including for animal welfare. Different stimuli (LPS or POLY I:C) induce different transcriptional responses, revealing virus and bacterial-induced transcriptional signatures (IL-1b, IL-6, IL-10, and TNF-a) in salmon and rainbow trout leukocytes [38]. Understanding the genes and pathways affected by different stimuli is essential for studying interactions between pathogens and farmed salmon. Functional PRRs, including toll-like receptors (TLRs), allow for rapid recognition of pathogens or PAMPs and initiate the innate and acquired immune response [23,24,25,26,39,40,41].

This study observed varied expression of these immune markers, indicating that *S. salar*, *O. kisutch* are more resistant to pathogens since a sustained expression was observed during the experimental period. However, in rainbow trout, the expression of all the immune markers decreased at 3 h. In addition, the exposure of head kidney culture cells to LPS and POLY I:C elicited a species-specific response in a time-dependent manner.

Our study extends these findings by demonstrating that TLR1, TLR-5, and TLR-8 are present in head kidney cells of *S. salar*, *O. kisutch*, and *O. mykiss* and also the upregulation of transcription of TLRs by LPS-stimulation. This is similar to the findings of Chettri et al., 2011, who observed that the presence of LPS induces TLR-5 transcription in rainbow trout head kidney leukocytes [34], demonstrating the immune stimulation.

The POLY I:C stimulation showed early (within a few hours) and late (last hours) response to the kinetic challenge in the three salmonids. Previous studies showed how granulosa cells express functional TLRs 1-9 and respond to POLY I:C [42,43,44]. In addition, kidney cells can recognize double-stranded RNA [45], ovarian surface epithelia express TLRs 2-5 [43] and cumulus cells express TLRs2, 4, 8, and 9 [46]. POLY I:C induces an antiviral response (TLR-3) in several fish species, such as *S. salar* [47], and the head kidney cells and liver cells of common carp (*Cyprinus carpio*) [48] and killifish (*Nothobranchius guentheri)* [49]. Pham et al. (2017) compared the *S. salar* heart endothelial cell line (ASHe) and the bulbous arteriosus of the fibroblast cell line (BAASf) for their responses to a viral mimic, POLY I:C, and to four RNA viruses: CSV, IPNV, VHSV Iva, and VHSV IVb [50].

A study published by Vargas-Chacoff et al. (2014) [51] showed that the Patagonian blennie *Eleginops maclovinus* increased type immunoglobulin M (IgM) levels in response to an intraperitoneal injection of total protein extract from bacterium *Piscirickettsia salmonis.* These results complement those observed in vitro, with high levels of IgM and MHC II expression in *S. salar* at 1, 24, and 48 h and at 1, 6, and 48 h in *O. kisutch* and a decrease in IgM at 3 and 24 h and in MHC II at 1 and 12 h, where LPS stimulated IgM expression at 6 h in *S. salar* and *O. mykiss* but at 0. 5, and 1 h in *O. kisutch*.

However, *O. mykiss* decreased gene expression of TLR-8, IgM, and MHC II at 48 h. This response was similar in *O. mykiss* infected with *F. psychrophilum*, having the lowest levels of mRNA in cell membrane-associated receptors such as TLRs in spleen. [13], in agree *Argyrosomus japonicas* presented a downregulation of MHC II at 12 and 48 h postinjection with POLY I:C in spleen [47]. 

Here we observed that MHC II expression increased significantly after LPS exposure in *S. salar* at 3 and 48 h and in *O. mykiss* at 0.5, 1, 6, and 12 h. However, in *O. kisutch*, we observed a sustained response at all time points in the gene expression of these genes as compared to the control group treated with both immunostimulants, observing a species-specific response differentially depending on the stimulus and time monitored.

## 5. Conclusions

The magnitude of all immune transcript markers was comparatively higher at 24 h in *S. salar* stimulated with LPS. In comparison, in *O. mykiss* and *O. kisutch*, peak responses were observed at 1 h and 48 h with both immunostimulants. Gene expression patterns of primary culture cells responded differentially depending on fish species and immunostimulants. This can be explained by the ability shown by cells from the primary kidney culture to recognize and respond to different immunostimulants such as LPS and POLY I:C.

Considering the significant economic losses incurred by the aquaculture industry due to bacterial and viral infection, cell cultures provide an alternative to in vivo experimentation. In addition, this method can provide reproducible results at a lower cost, and this tool can reduce the number of fish experimented upon, helping the 3Rs and animal welfare. The present study is the first demonstration that primary head kidney cells from the three most important Chilean salmonids differentially change the expression of immune genes in response to elements mimicking viruses and bacteria.

## Figures and Tables

**Table 1:** Primer sequences for immune system used in the experiments.

Primer	Nucleotide Sequences (5′→3′)	PCR Product Size	Efficiency HK (%)
MHCII Fw	CTACGAGTTCTACCCCAAACCCAT	102 bp	102.91
MHCII Rv	CAGTCGCTGTCAGCCAGTTCTT		
TLR1 Fw	CAACGCTATCTGATCCCCAAGCAA	114 bp	101.6
TLR1 Rv	AAAGCCGACGCTCAGTGTTTGT		
TLR5 Fw	TGGCTCACTACCAGCTGATGAA	112 bp	102.5
TLR5 Rv	AGCCGCTCATAAAACCACTC		
TLR8 Fw	TCCTGCAGAACTCTCACTTCCT	122 bp	101.9
TLR8 Rv	TCTGACCACATTCCTCAGGTTT		
IgMs Fw	TGAAAGACTTCTACCCGCATGAGG	124 bp	102.7
IgMs Rv	AACTGGCCATAAGCGGAAAAGG		
18s het Fw	GTCCGGGAAACCAAAGTC	116 bp	103.1
18s het Rv	TTGAGTCAAATTAAGCCGCA		

## Data Availability

Data Availability Statements are available by requirement.
